# Melody Valve Endocarditis Due to Rothia dentocariosa: A Diagnostic Challenge

**DOI:** 10.7759/cureus.8840

**Published:** 2020-06-26

**Authors:** Rahul Myadam, Christopher DeZorzi, Laura Schmidt, Peter Lin, Arthur I McGhie

**Affiliations:** 1 Internal Medicine, University of Missouri-Kansas City, Kansas City, USA; 2 Cardiology, University of Missouri-Kansas City School of Medicine, Kansas City, USA; 3 Cardiology, Saint Luke's Mid America Heart Institute, Kansas City, USA; 4 Pathology, Mayo Clinic, Rochester, USA; 5 Cardiology, Saint Luke's Mid-America Heart Institute, Kansas City, USA

**Keywords:** melody valve endocarditis, rothia dentocariosa, fluorodeoxyglucose positron emisison tomography

## Abstract

Recently, there have been several advances in the field of adult congenital heart disease, such as the percutaneous pulmonic valve implantation (PPVI) to treat right ventricular outflow obstruction. Complications from this technique are seldom but essential to understand. We present a case of a 37-year-old Caucasian male with complicated congenital heart disease, including prior Melody valve implantation, who presented to our hospital with recurrent episodes of pneumonia of two months duration. He was diagnosed with prosthetic valve endocarditis (PVE) from an unusual organism, *Rothia dentocariosa*. He eventually underwent surgical replacement of the infected valve. Our report is the first case of Melody valve endocarditis due to *Rothia dentocariosa* reported from the United States.

## Introduction

The percutaneous pulmonic valve implantation (PPVI) was developed as an alternative to the surgical reconstruction of the right ventricular outflow tract (RVOT) in the year 2000. The Medtronic Melody transcatheter pulmonary valve is one of the two commercially available valves for PPVI. The early and intermediate outcomes data have demonstrated excellent procedural success and freedom from RVOT intervention at rates of 98% at three years and 91% at five years [1,2]. However, one of the most important long-term complications of the Melody valve is infective endocarditis (IE), with the risk extending at least over the first three years after implantation [3]. It is important to fully understand the risk factors and microbiological causes of IE of the Melody valve, as about 52% of such cases lead to re-intervention and 8.7% of patients succumb to the infection [3]. We present an unusual case of an atypical organism *Rothia* *dentocariosa* causing Melody valve endocarditis.

## Case presentation

A 37-year-old Caucasian male presented to the ED with complaints of intermittent fevers, chills, and progressive dyspnea on exertion of two months duration. He was treated for pneumonia with antibiotics on three occasions and was hospitalized twice. He had a chipped tooth without any dental trauma a few days before the onset of symptoms. He had a past medical history of congenital bicuspid aortic valve with severe stenosis that was repaired at 19 years of age with the Ross-Konno procedure. At 25 years, he received a bio-prosthetic Mosaic pulmonary valve replacement due to pulmonic stenosis (PS). This valve was complicated by IE secondary to HACEK organisms and was treated with six weeks of intravenous antibiotics. However, he later had a #22 Melody valve implantation at age 31 due to valve insufficiency. An echocardiogram two months before the admission showed moderate PS with a peak gradient of 57 mmHg and mean gradient of 30 mmHg. On cardiac examination, there was a harsh, pan-systolic murmur in the left upper sternal border, with III/VI intensity, and no radiation to the carotid arteries. The jugular vein was distended up to the angle of the mandible. 2+ bilateral lower extremity edema was present up to the ankles. 

**Figure 1:**
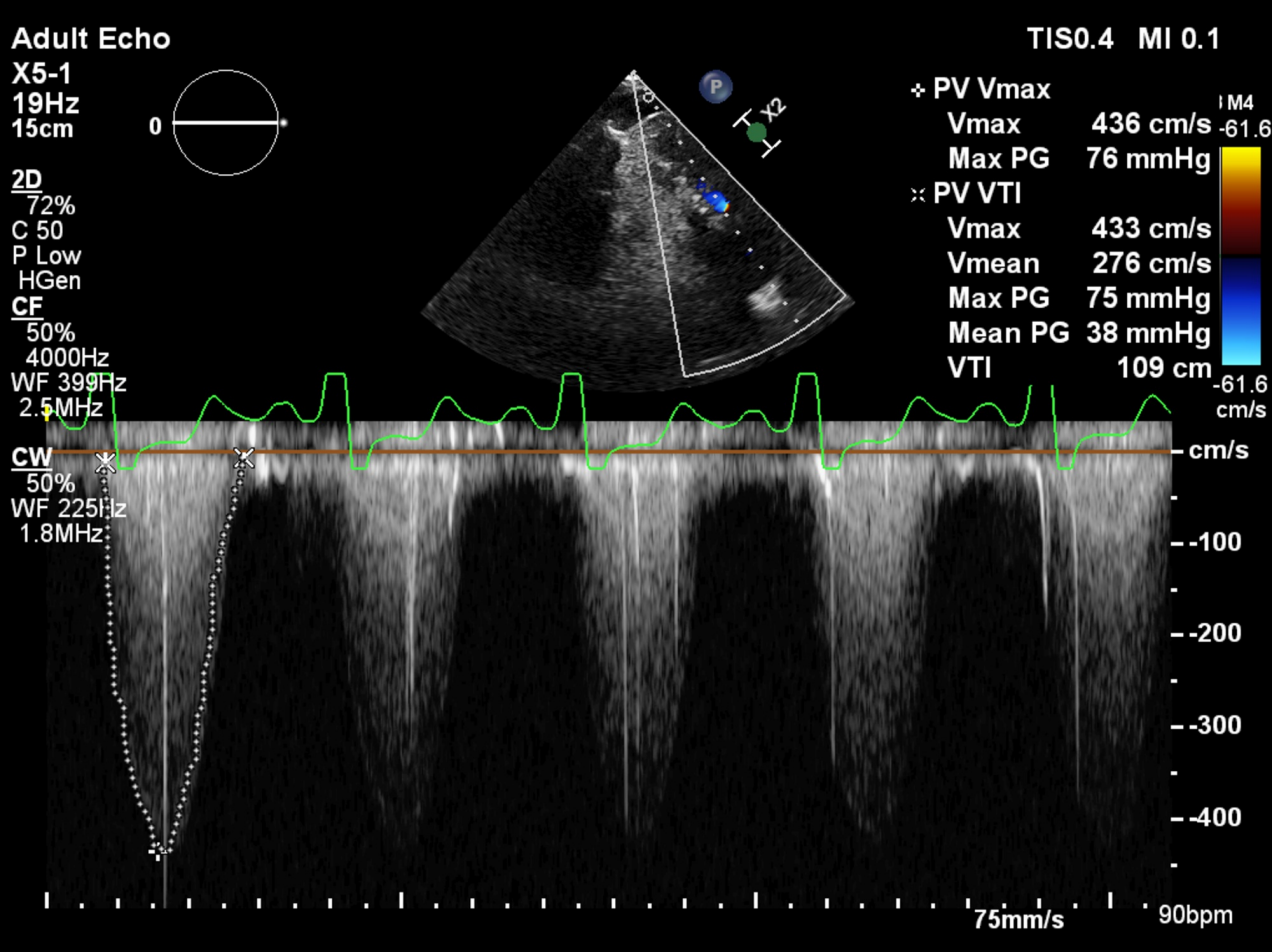
Transesophageal echocardiogram showing significant stenosis of the Melody valve

**Figure 2:**
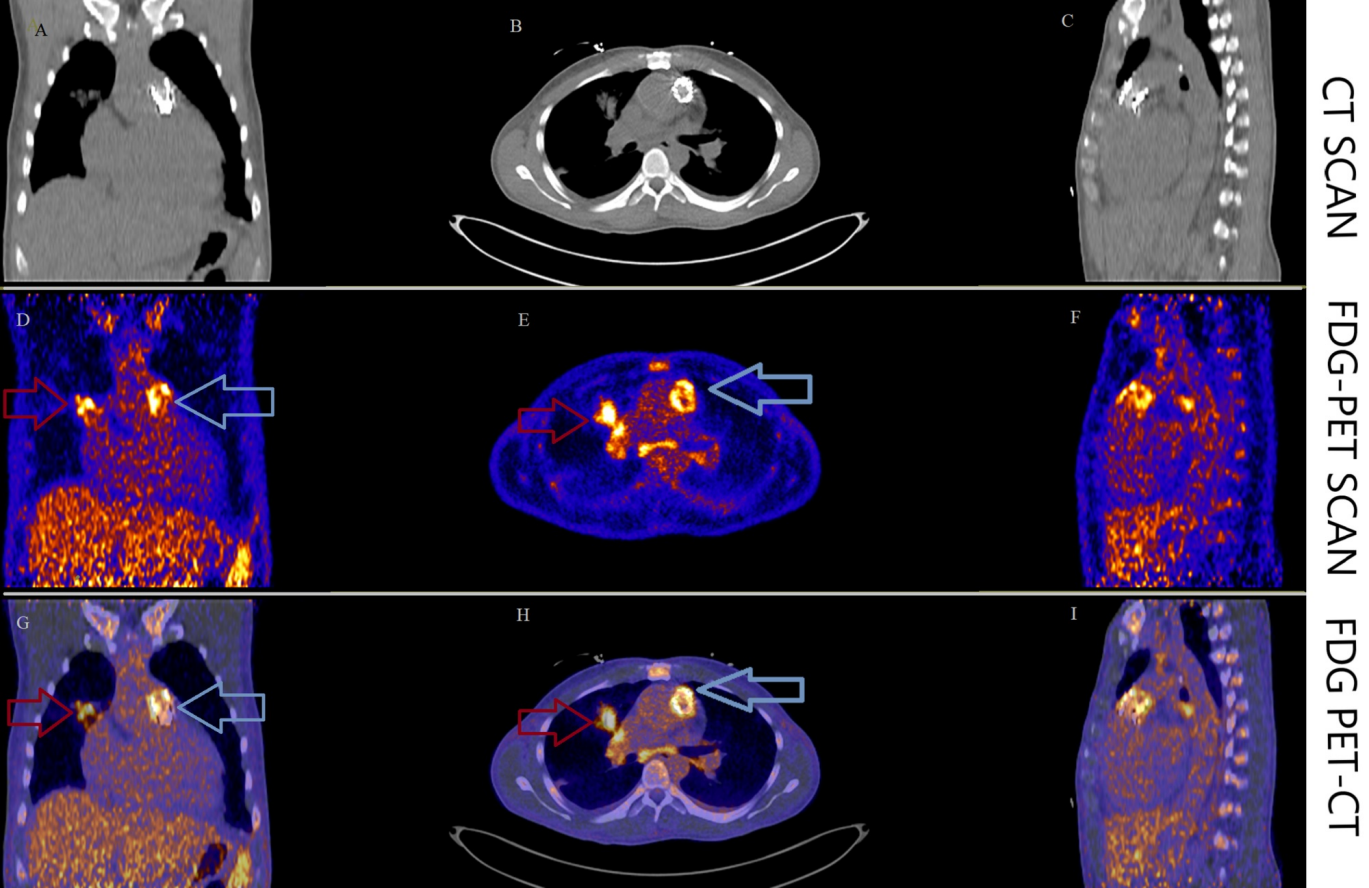
FDG PET/CT scan of the chest showing increased 18F uptake in the region of the Melody valve indicating PVE (blue arrows in panels D, E, G, H) and pulmonary septic embolus (red arrows in panels D, E, G, H). Coronal, transverse, and sagittal sections of the chest using CT scan (panels A, B, C), FDG-PET scan (panels D, E, F) and FDG PET-CT fusion (panels G, H, I). ^F^18 FDG uptake is noted in the region of the pulmonic Melody valve (blue arrows) consistent with the clinical diagnosis of infective endocarditis. Multiple areas of ^F^18 FDG uptake are noted in both lungs, one in the right hilar region (red arrows) and several in the subpleural region consistent with pulmonary septic emboli. FDG PET/CT: flourodeoxyglucose positron emission tomography-computerized tomography scan; PVE: prosthetic valve endocarditis

**Figure 3:**
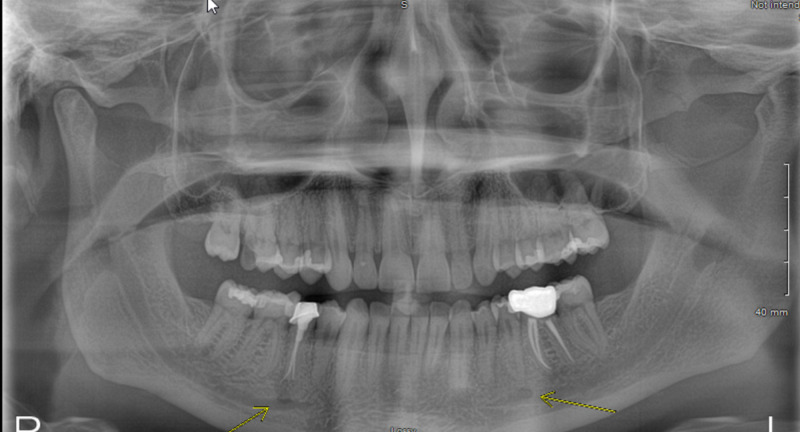
X-ray panorex orthopantogram showing dental abscesses in teeth #20 and #29 (see arrows)

The electrocardiogram showed sinus tachycardia and a new right bundle branch block. The laboratory data was notable for an N-terminal pro-brain natriuretic peptide (NT-pro BNP) of 8700 pg/ml. The transesophageal echocardiogram (TEE) showed severe PS with a peak velocity of 4.4 m/s, a mean gradient of 38 mmHg, peak gradient of 76 mmHg, and no pulmonic regurgitation (Figure 1). Small mobile echo-densities were present on the pulmonic valve (the largest vegetation measured ~ 0.8 cm in length). The right ventricle (RV) was severely dilated, with global hypokinesis, and severely reduced systolic function. The estimated RV systolic pressure was 85 mmHg. Of note, he also had an aneurysmal aortic root, measuring 5.0 cm at the sinuses of Valsalva. There was moderate regurgitation of the aortic and tricuspid valves. The blood cultures were positive for a gram-positive coccobacillus, later identified as *Rothia dentocariosa* in 2/2 bottles. An ^18^F-fluorodeoxyglucose positron emission computerized tomography scan (FDG PET/CT) was obtained due to the concern for endocarditis, which showed ^18^F uptake in the region of the Melody valve, confirming the diagnosis of prosthetic valve endocarditis (PVE) (Figure 2). There were also multiple areas of ^18^F uptake in both lungs, consistent with pulmonary septic emboli. An X-ray panorex orthopantogram showed odontogenic abscesses in teeth #20 and 29, with the latter associated with the chipped tooth (Figure 3).

**Figure 4:**
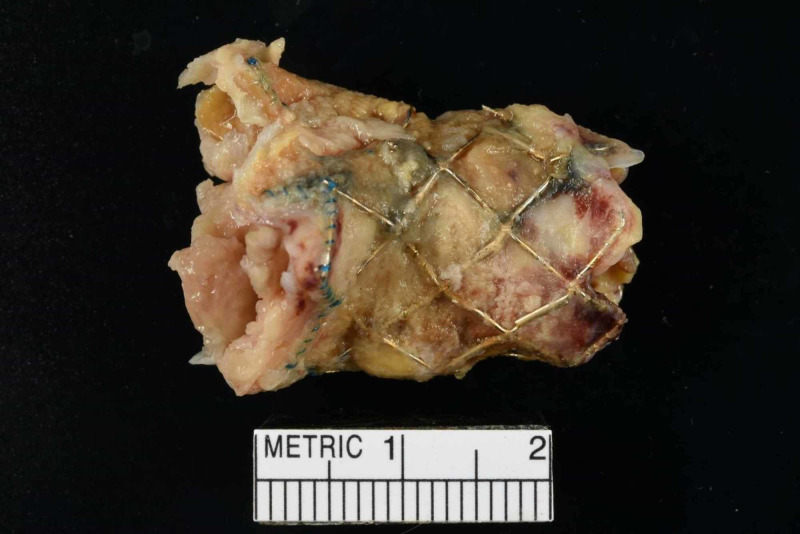
Gross photograph of the infected bio-prosthetic pulmonary valve with adherent friable soft tissue

**Figure 5:**
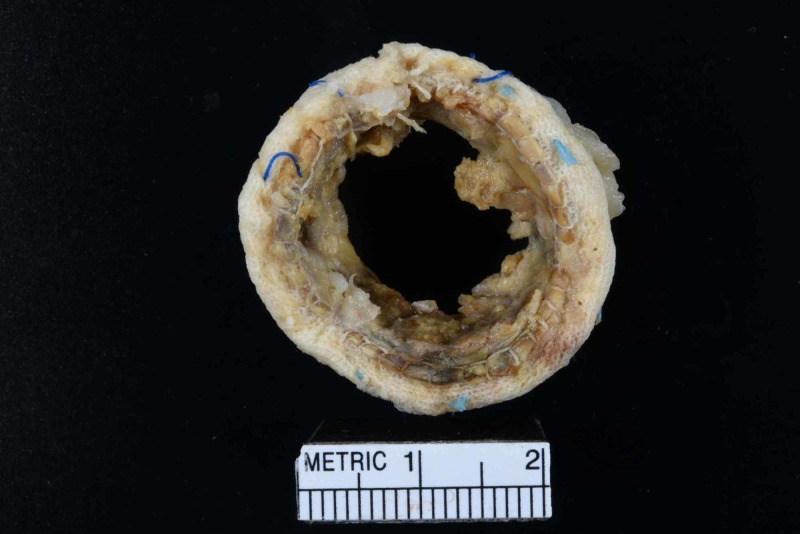
Gross photograph of the infected bio-prosthetic pulmonary valve with adherent friable soft tissue

**Figure 6:**
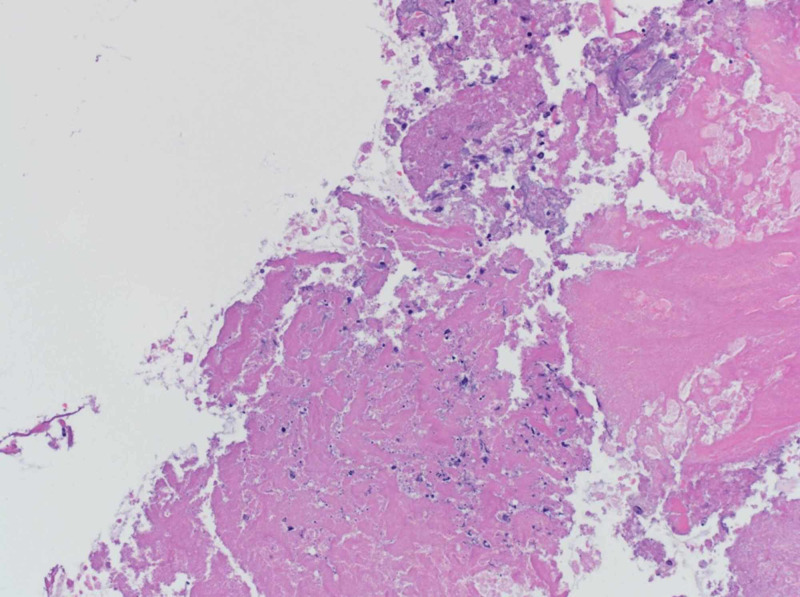
Hematoxylin and eosin-stained microphotograph of friable soft tissue of the Melody valve showing necrotic debris and minimal acute inflammation (20x objective)

**Figure 7:**
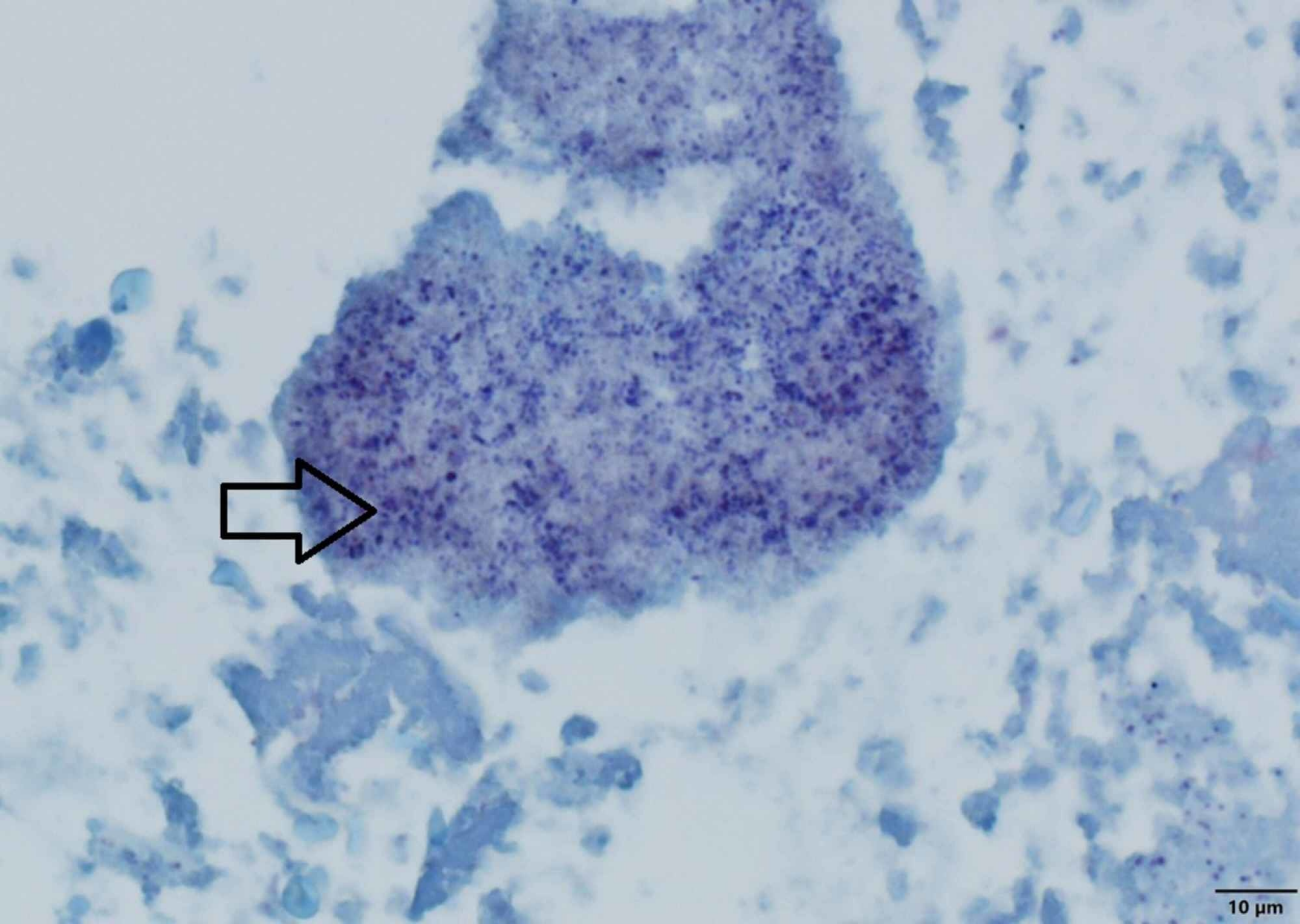
Gram-stained microphotograph of the Melody valve showing abundant Gram-positive bacteria within area of necrosis (see arrow) - 60x objective

**Figure 8:**
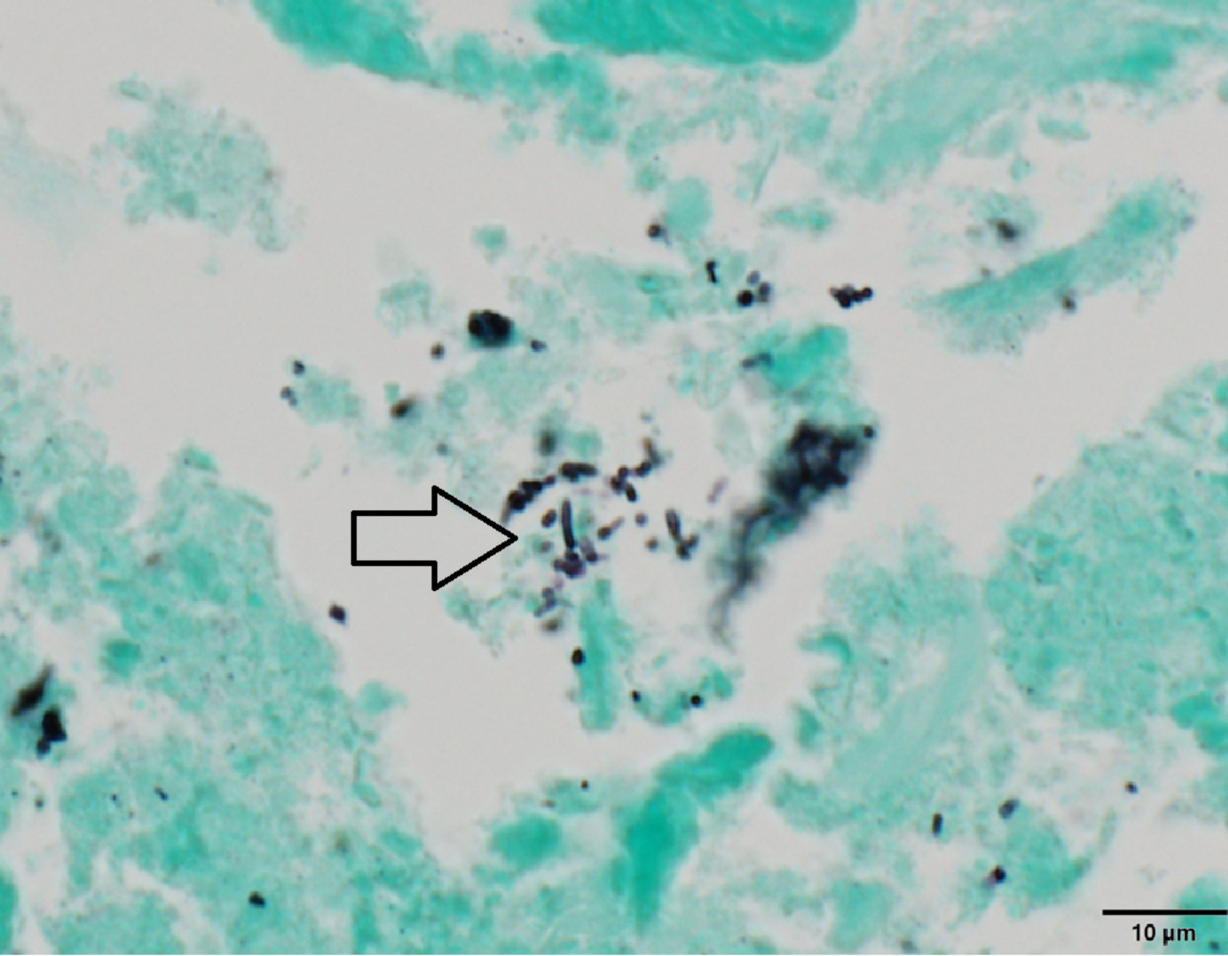
Gomori methenamine silver-stained microphotograph of the Melody valve showing coccobacilli consistent with Rothia dentocariosa (see arrow) - 60x objective

The patient had acute RV failure secondary to severe PS. Based on the modified Duke criteria, the patient was initially diagnosed with ‘possible PVE’. He had one possible major criterion (echo densities on the pulmonic valve were concerned for vegetations but fractured calcium could not be ruled out) and four minor criteria (predisposing cardiac condition, fever >38 C, septic pulmonary emboli, and microbiological cultures not meeting major criteria). The positive FDG PET/CT, especially in the setting of a worsening RVOT gradient, confirmed ‘definite PVE’. He was started on a continuous furosemide drip for diuresis, and a combination of vancomycin, and ceftriaxone for PVE. He remained febrile despite a week of intravenous antibiotics. The cardio-thoracic surgery team recommended acute surgical intervention. The patient was transferred to another facility with expertise in the Ross procedure complications where he underwent his third median sternotomy. He underwent an RV to PA conduit using a composite 25-mm rifampin-soaked On-X sinus of Valsalva mechanical conduit with a 28-mm rifampin-soaked proximal Hemashield Dacron graft, an aortic root replacement with a 23-mm rifampin-soaked On-X sinus of Valsalva mechanical valve conduit (with left and right coronary button transfer), and tricuspid valve repair with a 30-mm rifampin-soaked Carbomedics partial band. Intraoperatively, there was an infected bio-prosthesis within the RV to pulmonary artery (PA) conduit, with a large amount of clot and vegetation causing significant RVOT obstruction (Figures 4, 5). The histopathology of the infected valve showed necrotic debris with minimal acute inflammation (Figure 6). The Gram and Gomori methenamine silver stains showed numerous gram-positive coccobacilli morphologically consistent with *R. dentocariosa *(Figures 7, 8). Broad-range 16S polymerase chain reaction-based assay for bacterial identification confirmed the presence of *R. dentocariosa* DNA. He had an excellent recovery and was discharged home on warfarin for goal INR 2.5 to 3.5, given his mechanical aortic and pulmonary valves. Long term suppressive antibiotic therapy was not recommended. Prophylactic antibiotics were recommended with future dental procedures.

The patient was seen in the adult congenital heart disease clinic one month after his surgery. He was healing well, and a transthoracic echocardiogram showed the normal function of the mechanical aortic and pulmonary valves. The RV was mildly dilated with improved systolic function.

## Discussion

The Melody valve (Medtronic Inc, Minneapolis, MN) is one of the percutaneous pulmonary valves, that was approved by the US Food and Drug Administration in the year 2010. The annualized incidence rate for Melody PVE is reported to be 3.7% to 6.3% per patient-year [3]. The risk factors for IE of this valve include male gender, older age, cutaneous or dental infections, prior history of IE before PPVI, indwelling central line/dialysis catheter, and abrupt discontinuation of antiplatelets [4]. McElhinney et al. reported that *staphylococcal* species (43%) and viridans group *streptococcal* species (37%) were common causes, and other organisms such as coagulase-negative *staphylococcus*, HACEK bacteria, and nutritionally variant *streptococcus* were infrequent causes of Melody PVE [5]. Atypical organisms such as *Bartonella henselae, Aspergillus fumigatus, and Aerococcus viridans* have been rarely reported as the cause of Melody PVE [6-8]. We report the first case in the US of the Melody PVE caused by the *Rothia* spp. The *Rothia* spp. are part of the normal flora of the human oropharynx. They are reported to cause a wide range of conditions, especially in patients with profound neutropenia, malignancy, and indwelling vascular foreign body [9]. *R. dentocariosa* was first reported to cause human infection in 1975, in a 19-year-old woman with peri-appendiceal abscess [10]. Since then, several case reports of left-sided endocarditis caused by *R. dentocariosa *were reported in the literature [11-14]. However, the pulmonic involvement by *Rothia* was not reported previously. Typically, the *Rothia spp. *is considered a contaminant, and therefore the clinician needs to maintain a high index of suspicion for IE. Ramanan et al. reported that these organisms were susceptible to penicillin, ceftriaxone, meropenem, and vancomycin [9]. In our patient, occult dental abscesses due to *R. dentocariosa *were the source of the IE. The most recent US and European guidelines on the management of valvular heart disease recommend antibiotic prophylaxis before dental procedures in patients with prosthetic valves, including transcatheter-implanted prosthesis [15,16]. However, in our patient, there was no history of dental trauma or invasive procedure.

The role of novel imaging techniques such as an intracardiac echocardiogram, FDG PET/CT, and cardiac CT scan in the diagnosis and management of IE is evolving. The utility of the FDG PET/CT, when combined with modified Duke criteria to diagnose PVE is well known [17]. The FDG PET/CT scan provides higher sensitivity (91%-97%), better spatial resolution, and identifies extracardiac complications in PVE when compared to the echocardiogram [17]. The current European Society of Cardiology guidelines endorse using the FDG PET/CT as a major criterion [18]. However, the American College of Cardiology/American Heart Association guidelines on IE have not yet incorporated the use of these imaging modalities [19]. Nonetheless, the role of nuclear imaging of the Melody PVE is rarely described. Melekzadeh-Milani et al. reported using the FDG PET/CT to diagnose 'definite PVE' in five out of eight patients with a 'possible PVE' of the Melody valve based on the modified Duke criteria [4]. Our case provides further support for the use of the FDG PET/CT in the diagnosis and management of the Melody PVE.

Abdelghani et al. in a systematic review of 851 patients with the Melody valve implantation, reported that *streptococcal* infection and the lack of RVOT obstruction were associated with significantly better outcomes in patients with a Melody PVE [3]. However, despite having a high RVOT gradient, our patient survived. This is probably because of the subacute nature of the PVE. However, further research is needed to study the outcomes of Melody PVE due to atypical organisms such as *Rothia*.

## Conclusions

Despite good procedural outcomes, endocarditis of the Melody valve remains a major concern. The early diagnosis and treatment of the endocarditis prevents valve failure. The diagnosis is challenging in the setting of atypical organisms such as *Rothia species*, which are usually considered as contaminants. We provide support to the use of FDG PET/CT in these challenging cases of Melody PVE. 
